# Exact and Heuristic Multi-Robot Dubins Coverage Path Planning for Known Environments

**DOI:** 10.3390/s23052560

**Published:** 2023-02-25

**Authors:** Lin Li, Dianxi Shi, Songchang Jin, Shaowu Yang, Chenlei Zhou, Yaoning Lian, Hengzhu Liu

**Affiliations:** 1 College of Computer, National University of Defense Technology, Changsha 410003, China; 2 Artificial Intelligence Research Center (AIRC), Defense Innovation Institute, Beijing 100073, China; 3 Tianjin Artificial Intelligence Innovation Center (TAIIC), Tianjin 300456, China

**Keywords:** coverage path planning, Dubins robots, path planning

## Abstract

Coverage path planning (CPP) of multiple Dubins robots has been extensively applied in aerial monitoring, marine exploration, and search and rescue. Existing multi-robot coverage path planning (MCPP) research use exact or heuristic algorithms to address coverage applications. However, several exact algorithms always provide precise area division rather than coverage paths, and heuristic methods face the challenge of balancing accuracy and complexity. This paper focuses on the Dubins MCPP problem of known environments. Firstly, we present an exact Dubins multi-robot coverage path planning (EDM) algorithm based on mixed linear integer programming (MILP). The EDM algorithm searches the entire solution space to obtain the shortest Dubins coverage path. Secondly, a heuristic approximate credit-based Dubins multi-robot coverage path planning (CDM) algorithm is presented, which utilizes the credit model to balance tasks among robots and a tree partition strategy to reduce complexity. Comparison experiments with other exact and approximate algorithms demonstrate that EDM provides the least coverage time in small scenes, and CDM produces a shorter coverage time and less computation time in large scenes. Feasibility experiments demonstrate the applicability of EDM and CDM to a high-fidelity fixed-wing unmanned aerial vehicle (UAV) model.

## 1. Introduction

As one sub-problem of robot path planning, coverage path planning (CPP) aims to determine the optimal paths between the start and goal points to cover all regions while avoiding obstacles and satisfying intrinsic robot limitations [1,2]. CPP is common in several applications, including small-scale household tasks such as floor cleaning or lawn mowing and large-scale operations such as search and rescue and environmental monitoring [3]. Due to the limited sensing range, calculating speed, and energy supply, many practical coverage applications cannot be achieved by a single robot [4]. Thus, a series of multi-robot CPP (MCPP) algorithms have been proposed to improve coverage efficiency and enhance robustness. Meanwhile, MCPP faces the challenges of collaborative control, intelligent decision-making, and logistical management [1,5].

Real-world MCPP applications, such as aerial monitoring [6], marine exploration [7], and automatic farming [8,9], typically involve multiple aerial (fixed-wing aircraft), ground (wheel robots), and autonomous underwater/surface vehicles. These vehicles are typically governed by the Dubins vehicle model [10], which allows them to move at a fixed speed and turn with a limited turning radius. As the foundation of many practical applications, MCPP oriented towards Dubins robots (Dubins MCPP) has received growing attention in recent years. Thus, this paper focuses on the Dubins MCPP problem of known environments.

MCPP problem has been proven to be NP-hard [11]. Various MCPP works have been proposed to address the MCPP problem, and the related reviews can be found in [12,13,14]. Existing MCPP methods can be classified as exact or heuristic according to their accuracy [15]. Exact methods can provide the optimal solution for small-scale coverage applications. Heuristic methods are used to obtain a near optimal result for large-scale coverage applications since they often involve a great number of tasks.

Existing MCPP methods perform well, but they suffer from three issues. The first issue is that several exact methods always provide an accurate partition of the region. However, the accurate partition is not equivalent to optimal coverage paths for the MCPP problem. Second, heuristic methods always face the challenge of how to balance accuracy and complexity. Traditional heuristic methods represent coverage tasks as graphs and obtain an efficient result using graph-partition and tree-partition strategies. In the graph-partition strategy, all vertexes and edges are considered to achieve near-optimal results. However, its runtime increases since the search space increases exponentially with the number of vertexes [15]. The tree-partition strategy compresses the search space of the MCPP problem by pruning the edges of the graph, while the compressed search space reduces the runtime, it decreases the accuracy of the solution. The third issue is that many MCPP works have been proposed, but only a few studies have been conducted on the Dubins robot. As a curvature-constrained robot, the Dubins robot cannot recede and can only move at a fixed speed and with a bound curvature. Without consideration of robot kinematics, MCPP algorithms probably generate piece-wise paths that are only comprised of straight lines and sharp turns [16]. However, these paths are not feasible to follow for Dubins robots.

This paper presents two algorithms to address the Dubins MCPP problem. First, an exact Dubins MCPP (EDM) algorithm is proposed, formulating the Dubins MCPP problem as an MILP to produce the optimal Dubins coverage paths. Second, we present a heuristic approximate credit-based multi-robot Dubins MCPP (CDM) algorithm. CDM divides the region into multiple partitions by a tree-partition strategy and balances coverage tasks among partitions by the credit model [17]. The effectiveness of EDM and CDM was validated in comparison and feasibility experiments. In summary, the contributions of this paper are as follows:

- We present an EDM algorithm based on MILP, which provides the shortest Dubins coverage path by searching the entire solution space.
- We present a CDM algorithm, which ensures the task balance among robots by the credit model and reduces complexity by a tree-partition strategy.
- Extensive validations. (i) Comparison experiments with other exact and heuristic MCPP methods show that EDM provides the minimum coverage time in small coverage scenes, and CDM generates a shorter coverage time and less computation time in large coverage scenes. (ii) Feasibility experiments are conducted on a high-fidelity UAV model to validate the applicability of EDM and CDM.

The remaining of this paper is organized as follows. In Section 2, the related works are reviewed. Section 3 states the Dubins MCPP problem and presents a Dubins coverage framework. Section 4 and Section 5 describe EDM and CDM, including their ideas and implementation. The comparison and feasibility tests of EDM and CDM are presented in Section 6, followed by the paper’s conclusion.

## 2. Related Work

### 2.1. Exact and Heuristic MCPP Methods

As a hot topic in robotic research fields, MCPP has received increasing attention in recent years. The objective of MCPP is to find the shortest or fastest path to visit all points of a region while considering different missions and constraints. The constraints of MCPP include static factors (e.g., robot capability, region shapes, and obstacle locations) and dynamic factors (e.g., group goals and collaboration relationships) [18]. Existing works address the MCPP problem by using exact and heuristic algorithms. Exact algorithms guarantee an optimum solution, while heuristic algorithms seek to yield a good, but not necessarily optimal, solution. However, an exact algorithm takes much longer than a heuristic one to find an optimum solution to a difficult problem [19]. Thus, exact algorithms are suitable for small-scale applications, whereas large-scale coverage applications often use heuristics to achieve a suboptimal solution.

Exact MCPP algorithms use the MILP [20,21], branch and bound method [22], and dynamic programming method to obtain the optimum solution. Some exact algorithms precisely divide the region into *K* partitions and apply a single-robot coverage algorithm to each partition. For example, the work [20] transforms the MCPP problem into the MinMax balanced and connected *q*-partition problem ($BCP_{q}$) and presents an exact Milpflow algorithm to handle it. The Milpflow algorithm provides a precise partition of the region through a flow model and applies a single-robot CPP algorithm to each partition. However, the optimial partition is not equivalent to the optimal coverage paths. Other exact works build exact formations based on MILP to generate the shortest or fastest paths. For example, the works [4,15] produces the fastest coverage paths by building exact formulations. However, both works calculate the coverage time of a given region based on the scanning area rather than the coverage path. In fact, the coverage time of a given region depends on the time to cover the scanning area and the time to perform turns. Since turns are often costly for mobile robots, neglecting the cost of turns usually reduces the efficiency [23]. In order to minimize the cost of turns, the work [24] divides the region into cells and represents cells as a graph. The Dubins MCPP problem is formulated with the graph representation as a generalized traversal salesman problem (TSP). The exact coverage path is then obtained by applying the GTSP solver. Unfortunately, the work [24] is only applicable to a single robot. Heuristic MCPP algorithms usually decompose the region into cells and represent them in a graph. With the graph representation, graph-partition and tree-partition strategies are utilized to divide the graph into multiple parts. Each part corresponds to one robot. The graph-partition strategy takes all information of the graph into account to obtain a (near-)optimal result. For example, to address the area patrolling problem of heterogeneous robots, the work [25] utilizes the auction algorithm to assign appropriate tasks to robots. Although the auction algorithm has the advantage of low complexity, its greedy strategy leads to local-optimal allocation. The authors in [17] extend the traditional market-based methods and propose a credit-based task allocation (CTA) algorithm. The CTA algorithm balances the tasks among robots by a credit model and reduces complexity by transforming a multi-objective optimization problem into a set of single-objective optimization problems. However, the CTA algorithm is unsuitable for coverage applications relying on Morse or BCD decomposition since it assumes that coverage tasks are uniform grids. In [3], two heuristics algorithms are presented to address the MCPP problem for known environments. The first algorithm calculates the Eulerian tour of visiting all tasks and produces *k* sub-tours by a k-postman approximation algorithm. The second one uses a greedy approach that divides the area into equal regions, covering each region with a single robot. Although the graph-partition strategy performs well, it has a long runtime for graphs with a significant number of vertexes and edges.

The tree-partition strategy reduces the runtime by deleting edges from the graph. However, some optimality is sacrificed since the search space shrinks after edge deletion. In [26], the authors proposed a spanning tree coverage (STC) algorithm for single robots. The STC algorithm incrementally builds a virtual tree and navigates the robot around the tree to achieve complete coverage. The work [27] extends the STC algorithm and presents a multi-robot STC algorithm, which reduces coverage time by two times while no repeated tasks are generated. Nevertheless, it cannot guarantee an optimal result with the increase in robots. Literature [28] proposed a polynomial-time algorithm that assigns tasks to *k* robots by finding a weighted tree of *k* (k= number of robots) covering all nodes. The polynomial-time algorithm ensures that its coverage time is eight times the optimal coverage time. However, it assumes that the trees can be overlapped. The genetic algorithm was also used to solve the tree-partition problem due to its excellent performance. In the genetic algorithm, each individual is composed of a forest of non-intersecting trees, and the population evolves to find the (near-)optimum. For example, the work [20] presented a genetic algorithm based on tree partitioning and evolution. The genetic algorithm can handle graphs with up to 3000 nodes. Ref. [29] presents an algorithm called mofint for finding the least number of robots within a time limit. The mofint algorithm transforms the time-limit version of MCPP into a bi-objective optimization problem and applies a multi-objective genetic method. Although genetic algorithms perform well, they often produce local optimal solutions due to their evolutionary operations.

### 2.2. Dubins Coverage

The exact and heuristic MCPP methods provide optimal or near-optimal paths that visit all points of the region. However, due to their lack of consideration of robot kinematics, several MCPP methods could not guarantee the path’s curvature continuity. Curvature discontinuities threaten robot safety and degrade the robots’ dead-reckoning abilities [30,31]. Thus, the construction of feasible and smooth paths has received much attention in robotic research fields [16].

The Dubins path [10] provides the shortest path for robots with a single forward speed and a maximum turning radius in open areas. As Dubins paths can be expressed analytically and are quickly computed, a series of Dubins coverage methods based on them were presented. Dubins coverage has numerous practical applications, such as automated agriculture [32], search and rescue, and seabed inspection. For example, the authors in [33] presented a coverage algorithm for a fixed-wing unmanned aerial vehicle (UAV). The coverage algorithm breaks the region into multiple subcells and produces the Eulerian circuit with minimal path repetition. The effectiveness of the proposed algorithm [33] has been validated in field trials. The authors in [24] modelled the Dubins coverage problem into an generalized traversal salesman problem (GTSP). The coverage path with the lowest non-working travel is obtained by transforming the GTSP into an asymmetry traversal salesman problem (ATSP). By re-setting the path cost between two points separated by obstacles, the work [34] extended [24] to non-convex environments. The work [35] proved that the optimal Dubins coverage problem is NP-complete and presents a coverage algorithm for a single Dubins robot. In [3], the authors presented two heuristics methods called DCRC and DCAC for addressing the CPP problem with multiple Dubins vehicles. DCRC generates an optimal Hamiltonian path and uses route clustering to divide the path into *K* sub-paths. DCAC divides the area into multiple partitions and applies the single-robot Dubins solver [35] to each sub-area. The simulation results in [3] show that DCRC has better performance than DCAC.

## 3. System Overview

This section describes related definitions of the Dubins MCPP problem and presents a Dubins coverage framework.

### 3.1. Problem Statement

We assume to have *K* homogeneous Dubins robots to perform the coverage task. All robots constitute a robot set $R=\{r_{1},\ldots ,r_{K}\}$. The Dubins robots in *R* are equipped with the same task sensor for specific tasks (e.g., cleaning the floor or detecting objects). The task sensor can cover a rectangular area of $w_{1}$ in width. All robots start from the same starting point $p_{s}$ and travel at a fixed speed *s* with a minimum turning radius *r*.

**Figure 1:**
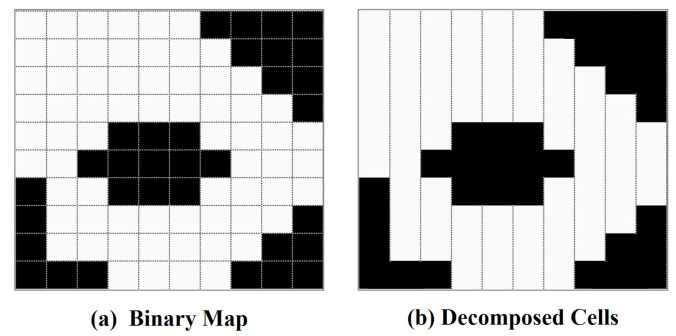
Decomposing the mission environment into cells.

The mission environment is assumed to be known and has been represented as a binary map. In this binary map, cells with values of 0 or 1 represent obstacles or allowed areas, respectively. To avoid being restricted to one kind of robot model, classical Dubins approaches [3,35] assume that obstacles are areas that are not necessarily covered but can be crossed. Similarly, this paper assumes that the robot can cross the obstacle. The semi-BCD decomposition [35] method is used to divide the region into *N* rectangular cells. All cells form the set of cells $C=\{c_{1},\ldots ,c_{n},\ldots ,c_{N}\}$, where $c_{n}$ represents the *n*-th cell in *C*. Each cell has a width of $w_{1}$, and its height depends on the boundary of the region and obstacles. Figure 1 represents an example of area decomposition.

The objective of MDCPP is to produce a path for each robot so that every point of the allowed areas is covered at least by one robot. Since all robots have the same kinetic constraints, an efficient solution is to minimize robot path lengths while equally distributing robot workloads. Hence, Dubins MCPP can be viewed as a MinMax problem, i.e., to minimize the maximum cost of all robots.

### 3.2. System Overview

**Figure 2:**
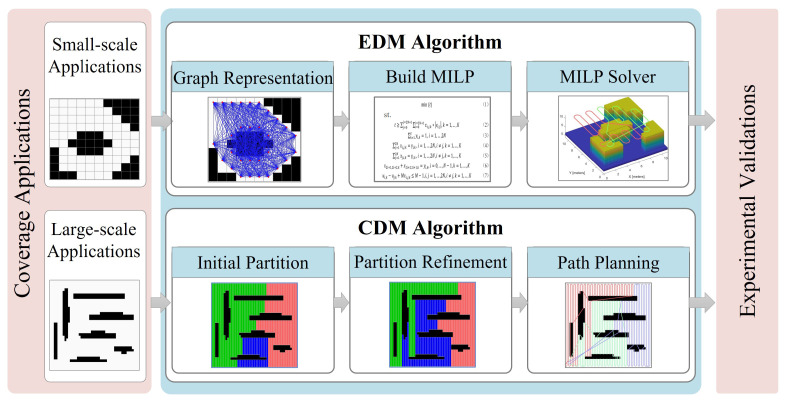
An overview of the Dubins coverage framework.

This paper presents a Dubins coverage framework to address the Dubins MCPP problem. As shown in Figure 2, the framework comprises coverage applications, Dubins MCPP methods, and experimental validations. The component of the Dubins MCPP methods consists of an EDM algorithm and a heuristic CDM algorithm. The EDM algorithm represents coverage tasks as a graph and proposes an exact formation based on MILP. The MILP solver is used to produce the optimal Dubins coverage path by thoroughly searching the solution space. The CDM algorithm divides the region into *K* sub-areas through initial partition and partition refinement modules. The single-robot Dubins solver [35] is then employed in each sub-area. More details of EDM and CDM are presented in Section 4 and Section 5.

## 4. Exact Dubin Multi-Robot CPP (EDM) Algorithm

Exact methods either provide an accurate partition or produce coverage paths without considering the turning cost of the robot covering a given region, resulting in a non-optimal coverage path. This paper presents an EDM algorithm to plan coverage paths. The EDM algorithm consists of two steps: graph representation and build MILP. The former step is to calculate the Dubins paths for covering coverage cells and turning from one cell to another. All Dubins paths will be represented as a connected graph. The latter step generates an exact formulation based on MILP to obtain the shortest Dubins coverage path.

### 4.1. Graph Representation

**Figure 3:**
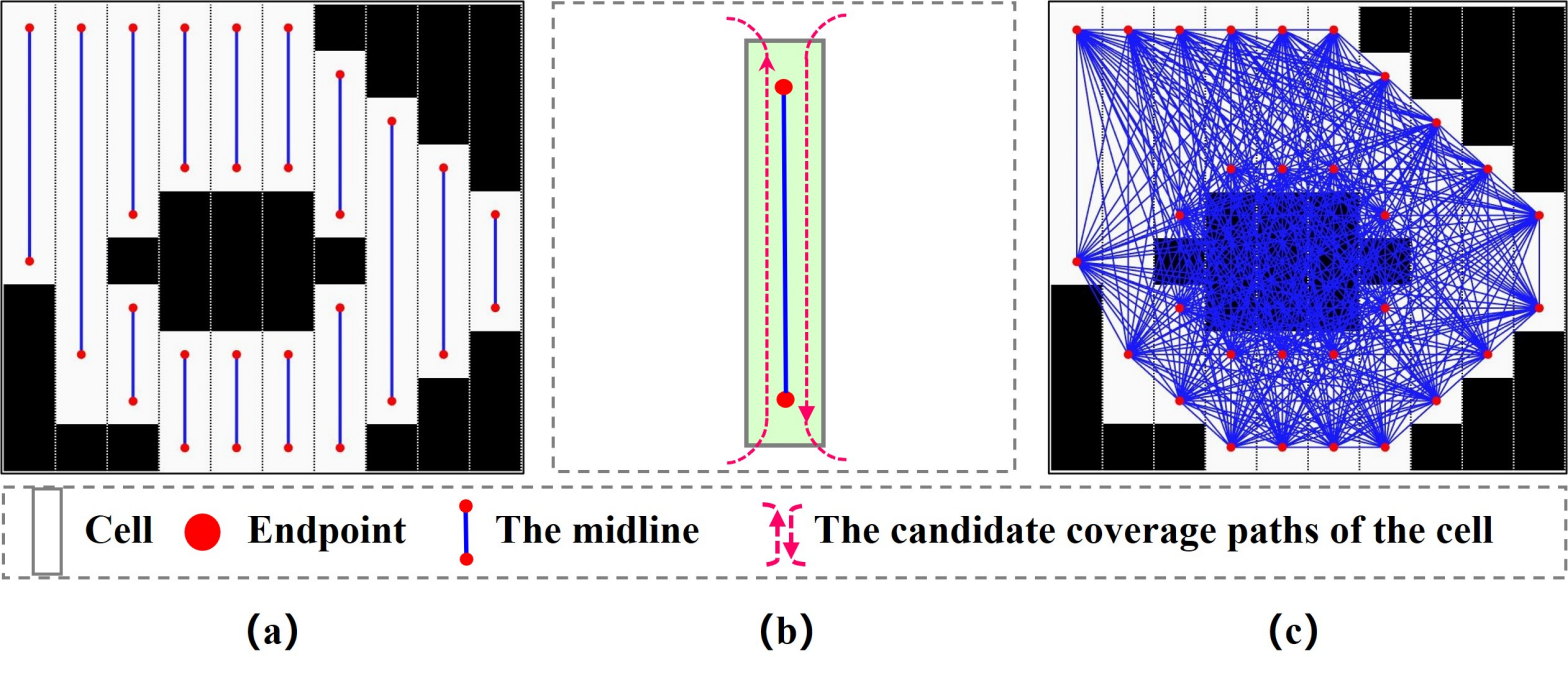
Graph representation. (**a**) Endpoints of each cell. (**b**) Coverage paths of each cell. (**c**) Graph.

Classical offline coverage methods decompose the region into cells and represent cells into a graph [5]. With the graphical representation, the MCPP problem is transformed into a TSP or Chinese Postman Problem to obtain the fastest or shortest path [3,24]. As most offline MCPP methods do, the EDM algorithm divides the mission environment into a set of cells (i.e., *C*). Each cell in *C* consists of two endpoints and a line segment connecting them. As shown in Figure 3a, the robot can either enter the cell from the top endpoint and cover it from the top down or enter the cell from the bottom endpoint and cover it from the bottom up. *N* cells of *C* correspond to 2N endpoints, which constitute the set of endpoints $P=\{p_{1},\ldots ,p_{2N}\}$. Each pair of endpoints $p_{2n+1}$ and $p_{2n+2}$ indicate the upper and lower endpoint of $c_{n},0\leq n\leq N-1$, respectively. All endpoints in *P* are represented as a connected graph G=(V,E), where *V* and *E* refer to vertex and edge sets, respectively. *V* consists of 2N+1 vertexes, where the first vertex $v_{0}$ corresponds to the starting point $p_{s}$, and other vertexes $v_{n},n=1,...,2N$ represent the *n*-th endpoint $p_{n}$ in *P*. Each edge $e_{i,j}\in E$ indicates the Dubins path between the vertex $v_{i}$ and $v_{j}$. Different from the graph presented in [3,5,24], *E* consists of the Dubins paths for the robot turning and covering the cell.

**Figure 4:**
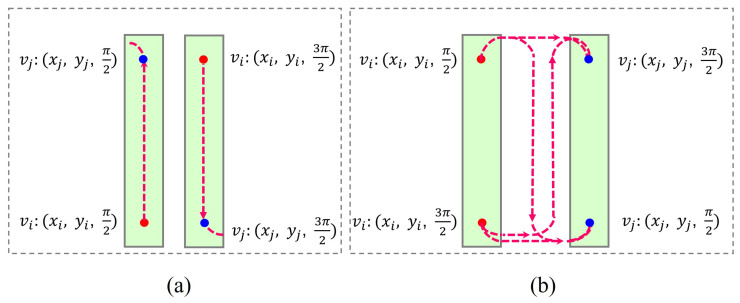
The start pose $v_{i}:\left(x_{i},y_{i},\theta_{i}\right)$ and target pose $v_{j}:\left(x_{j},y_{j},\theta_{j}\right)$. (**a**) $v_{i}$ and $v_{j}$ belong to different cells. (**b**) $v_{i}$ and $v_{j}$ belong to the same cell.

The edge between $v_{i}$ and $v_{j}$ represents the Dubins path from the start pose $v_{i}:\left(x_{i},y_{i},\theta_{i}\right)$ to the target pose $v_{j}:\left(x_{j},y_{j},\theta_{j}\right)$. The *x*/*y* coordinates $\left(x_{i},y_{i}\right)$ and $\left(x_{j},y_{j}\right)$ are fixed, but the angle $\theta_{i}$ and $\theta_{j}$ depends on $v_{i}$ and $v_{j}$’s relative positions. There are two cases for $\theta_{i}$ and $\theta_{j}$. In the first case, $v_{i}$ and $v_{j}$ belong to the same cell. Suppose that $v_{i}$ is the lower endpoint of the cell. The robot enters the cell from $v_{i}$ and covers the entire cell from bottom to top. Thus, $\theta_{i}$ and $\theta_{j}$ are set as $\frac{\pi }{2}$; alternatively, $\theta_{i}=\theta_{j}=\frac{3\pi }{2}$. The second case is that $v_{i}$ and $v_{j}$ belong to different cells. $\theta_{i}$ will be set to $\frac{3\pi }{2}$ if $v_{i}$ is the lower endpoint of the cell (i.e., the robot leaves the cell from its lower endpoint); otherwise, $\theta_{i}=\frac{\pi }{2}$. Similarly, $\theta_{j}$ will be set to $\frac{\pi }{2}$ if $v_{j}$ is the lower endpoint of the cell (i.e., the robot enters the cell from its lower endpoint); otherwise, $\theta_{j}=\frac{3\pi }{2}$. Figure 4 shows an example of how to calculate the angle. After determining the start and target pose, the Dubins path between $v_{i}$ and $v_{j}$ is calculated. The length of the Dubins path is set as the weight of $e_{i,j}$.

### 4.2. Build MILP

The optimal coverage aims to find *K* tours that start and end at the depot $p_{s}$, so every point of the allowed areas is visited, and the maximum cost of *K* tours is minimized. Thus, EDM models the Dubins MCPP problem as an MILP with the MinMax objective of minimizing the longest path cost among *K* robot paths. When *t* is defined as the cost of the longest Dubins path. The objective is

(1) $$\begin{array}{c} min(t) \end{array}$$

s.t.,

(2) $$\begin{array}{c} t\geq \sum_{j=0}^{2N+1}\sum_{i=0}^{2N+1}x_{i,j,k}\left|e_{i,j}\right|,k=1,\ldots ,K \end{array}$$

(3) $$\begin{array}{c} \sum_{k=1}^{K}y_{i,k}=1,i=1,\ldots ,2N \end{array}$$

(4) $$\begin{array}{c} \sum_{j=1}^{2N}x_{i,j,k}=y_{i,k},i=1,\ldots ,2N,i\neq j,k=1,\ldots ,K \end{array}$$

(5) $$\begin{array}{c} \sum_{j=1}^{2N}x_{j,i,k}=y_{i,k},i=1,\ldots ,2N,i\neq j,k=1,\ldots ,K \end{array}$$

(6) $$\begin{array}{c} \begin{array}{cc} x_{2i+1,2i+2,k} & +\;x_{2i+2,2i+1,k}=y_{i,k}\\ \; & i=0,\ldots ,N-1,k=1,\ldots ,K \end{array} \end{array}$$

(7) $$\begin{array}{c} \begin{array}{cc} u_{i,k} & -\;u_{j,k}+Mx_{i,j,k}\leq M-1\\ \; & i,j=1,\ldots ,2N,i\neq j,k=1,\ldots ,K \end{array} \end{array}$$

(8) $$\begin{array}{c} x_{i,j,k}\in \left\{0,1\right\},y_{i,k}\in \left\{0,1\right\},\forall i,j,k \end{array}$$

where $x_{i,j,k}=1$ represents that the robot $r_{k}$ visits vertex *j* immediately after vertex *i*; otherwise, $x_{i,j,k}=0$. $y_{i,k}=1$ indicates that the robot $r_{k}$ visits vertex *i*; otherwise, $y_{i,k}=0$. Equation (3) states that each vertex should be visited exactly once. Equations (4) and (5) ensures that once a robot visits a vertex, it must also depart from the same vertex. Equation (6) ensures that two endpoints of a cell should be traversed by sequence. Equation (7) is the MTZ-based sub-tour elimination constraints [36]. The MILP is an extension of the asymmetry multiple travelling salesman problem (MTSP).

### 4.3. Pseudo-Code of the EDM Algorithm

Algorithm 1 shows the pseudo-code of the EDM algorithm. It inputs the region (A), the robot is set (*R*) and the starting point ($p_{s}$) and outputs *K* coverage paths $\left\{P_{1},\ldots ,P_{K}\right\}$. First, *K* coverage paths are initialized as empty (Line 1), and the region A has been divided into a set of cells *C* (Line 2). The cell set *C* is represented as a graph *G* (Line 3), and the cost matrix associated with *G* is calculated (Line 4). EDM models the Dubins MCPP problem as an MILP (Line 5) and utilizes the MILP Solver [37] to obtain the traversal sequence $\left\{T_{1},\ldots ,T_{K}\right\}$ (Line 6). Coverage paths are calculated according to $\left\{T_{1},\ldots ,T_{K}\right\}$ (Line 7).

*Computational Complexity*: Let *M* be the number of vertexes in *G*. The cost matrix can be calculated in O$\left(M^{2}\right)$ times. The asymmetrical MTSP can be transformed into an asymmetrical TSP, which tasks O$\left(M^{3}\right)$ times in the worst case [38]. Thus, the overall complexity of EDM is O$\left(M^{3}\right)$.
**Algorithm 1** EDM Algorithm**Input**: A,R, $p_{s}$**Output**: $P_{1},\ldots ,P_{K}$
  1:  Initialize: $P_{1},\ldots ,P_{K}\leftarrow \emptyset$;

  2:  C← Area_Decomposition(A);

  3:  G← Graph_Representation($C,p_{s}$);

  4:  CostMatrix← calculate the cost between points in *G*

  5:  Build_MILP(*G*); // Equations (1)–(7)

  6:  $\{T_{1},\ldots ,T_{K}\}\leftarrow$ MILP_Solver;

  7:  $\{P_{1},\ldots ,P_{K}\}\leftarrow$ Dubins_Solver($\left\{V_{1},\ldots ,V_{K}\right\}$);

  8:  **return**
$P_{1},\ldots ,P_{K}$


## 5. Heuristic Credit-Based Dubin Multi-Robot CPP(CDM) Algorithm

EDM algorithm provides an effective weapon to plan the exact coverage paths for small-scale coverage applications. However, several coverage applications involve a large number of coverage tasks and allow for near-optimal results. Therefore, this paper presents a heuristic CDM algorithm consisting of three components: initial partition, partition refinement, and path planning. The initial partition component utilizes the regional growth strategy to divide the region into *K* sub-areas. The partition refinement component balances *K* sub-areas by a tree-partition strategy, and the path planning component employs the single-robot Dubins solver [35] to each sub-area.

### 5.1. Initial Partition

**Figure 5:**
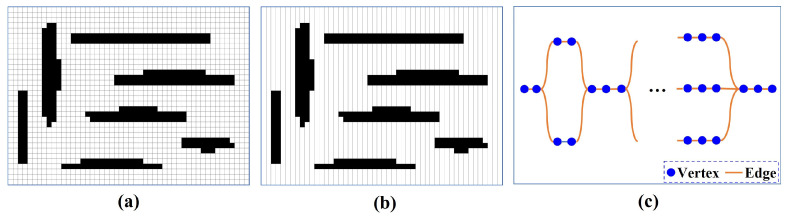
Graph representation. (**a**) The input map. (**b**) Coverage cells. (**c**) The connected graph.

As mentioned in Section 3.1, the mission environment has been divided into a set of cells *C*. The initial partition component represents *C* as a connected graph G1={V1,E1}, where the vertex represents the cell, and the edge represents the common border between cells. Figure 5 shows an example of the graphical representation. The region growth strategy based on the credit model [17] is used to divide the region (i.e., the graph G1) into *K* partitions.

In the credit model, *K* partitions act as traders in the virtual economy. Coverage cells are tradable commodities with measurable values. Additionally, a virtual bank is introduced. Traders can open credit accounts with a balance equal to $\frac{w(V1)}{K}$. The virtual bank maintains accounts and manages assets for traders. Each trader can borrow from the bank (without interest) if their account balance reaches zero. When a trader buys a cell $v_{t}$, its account balance is reduced by $|v_{t}|$, where $|v_{t}|$ represents the area of the cell $v_{t}$. On the other hand, if a trader sells a cell $v_{t}$, the account balance increases by $|v_{t}|$. Traders continue to buy cells, and the corresponding account balance decreases.

**Figure 6:**
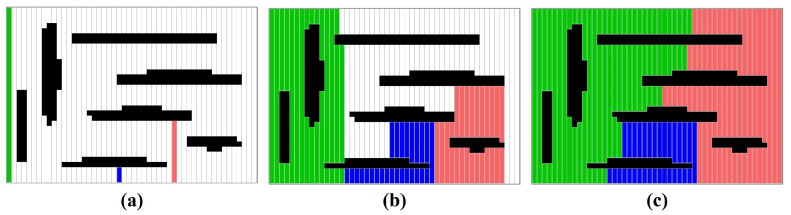
An example of the initial partition for three robots. (**a**) The seeds of three partitions. (**b**) Every partition grows around its seed. (**c**) The initial partition.

The initial partition component uses the regional growth strategy to divide the region into *K* partitions. First, cells in *C* are sorted in increasing order by the x-coordinate, followed by the *y*-coordinate, resulting in a sequence of cells *S*. The *K* cells, distributed at equal intervals in *S*, are set as the seeds of the *K* partitions. Second, every partition alternately buys cells and grows around the seed as the number of bought cells increases. All partitions become larger and larger until all cells in G1 have been purchased. In this case, G1 has been divided into *K* partitions. Figure 6 shows an example of the region growth strategy.

### 5.2. Partition Refinement

Due to complex obstacles, the initial *K* partitions may not be balanced. In order to obtain a balanced result, the partition refinement component reallocates tasks among partitions by way of task transactions. Each task transaction is performed in three steps. The first step is to determine the two partitions for the task transaction. The *k*-th partition (i.e., $V_{k}$) with the largest account balance is set as the buyer, i.e., the partition that receives tasks. Let ADV be the set of partitions adjacent to $V_{k}$. The partition u∈ADV that has the greatest difference with the account balance of $V_{k}$ becomes the seller, i.e., the partition that dispatches tasks. found(k) and found(u) represents the account balance of the seller and buyer, respectively.

**Figure 7:**
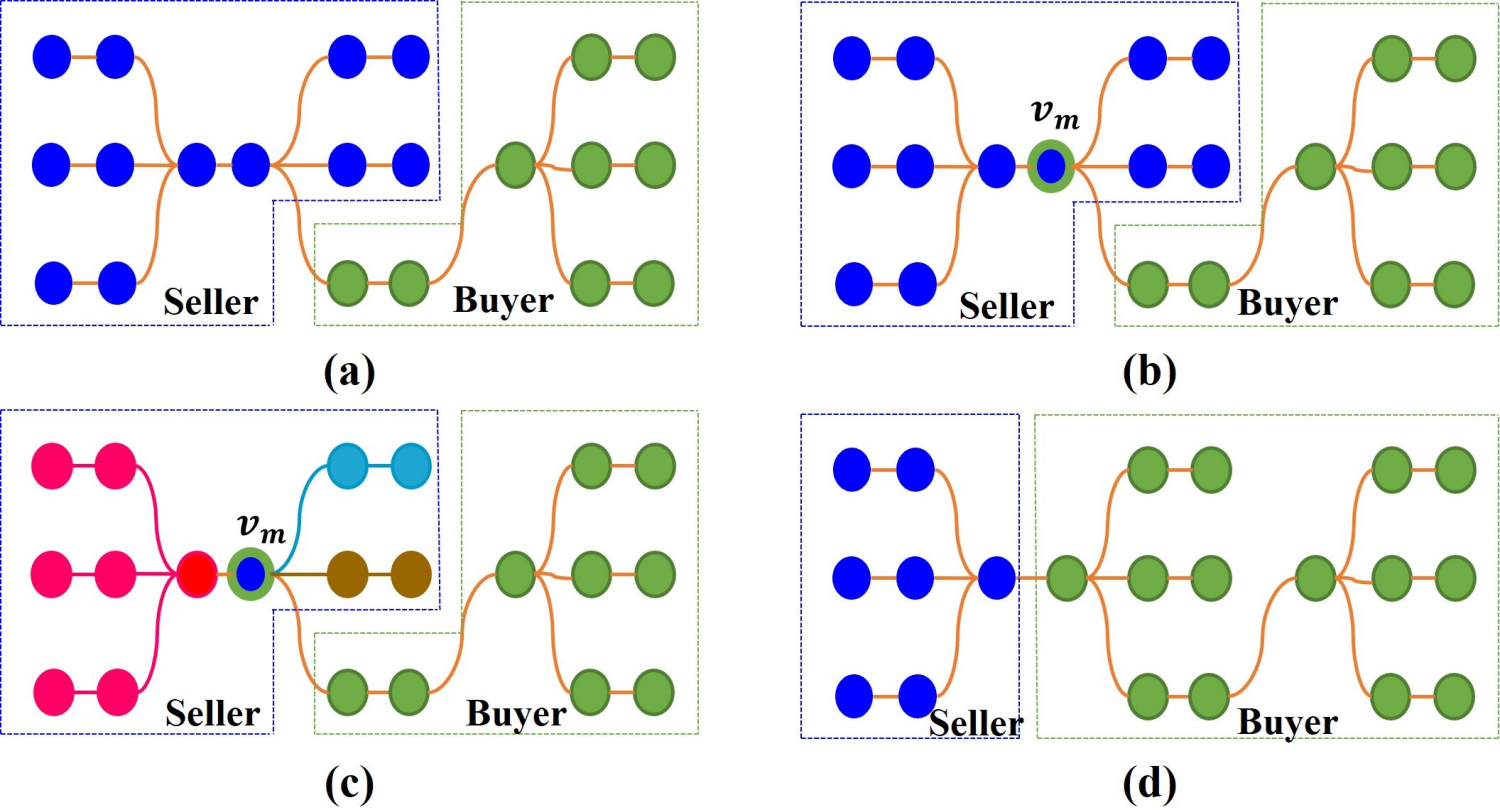
An example of the tree-partition strategy. (**a**) The graphs of the seller and buyer. (**b**) The adjacent vertex $v_{m}$. (**c**) Three sub-trees with the root $v_{m}$ in the seller. (**d**) The buyer and seller after the task transaction.

The second step is to decide which tasks the seller and buyer will trade. Suppose that $AC\in V_{u}$ is the cell set that shares a common border with $V_{k}$. The cell $v_{m}$ with the biggest weight in AC is selected for the candidate trade task EV. There are two possible cases, depending on the connection between $v_{m}$ and $V_{u}$. In the first case, $v_{m}$ is not the cut point of $V_{u}$. The trade task EV is set as $v_{m}$ since both seller and buyer remain connected after the task transaction. The second case is that $v_{m}$ is the cut point of $V_{u}$ (i.e., removing $v_{m}$ disconnects $V_{u}$). The seller $V_{u}$ becomes disconnected if it sells $v_{m}$ to $V_{k}$. However, disconnected partitions cause robot collisions and complicate robot control [29]. Thus, the depth first search (DFS) method is utilized to find the *Q* sub-trees $\left\{V_{u,1},\ldots V_{u,Q}\right\}$ in $V_{u}$ whose root nodes are $v_{m}$. $V_{u,i}$ and $V_{u,j},i\neq j,i,j=1,\ldots ,Q$ will be disconnected if $v_{m}$ is removed from $V_{u}$. In order to maintain its connectivity, the seller $V_{u}$ needs to reserve one sub-tree and set the other tasks as trade tasks. In order to determine which sub-tree the seller retains, a transaction index is defined, which quantifies the balance between the buyer and seller’s tasks. Suppose the seller reserves the *q*-th sub-tree $V_{u,q}$. The seller and buyer will be updated to $V_{k^{\prime }}=V_{k}\cup \left(V_{u}-V_{u,q}\right)$ and $V_{u^{\prime }}=V_{u,q}$, respectively. The trade index $\delta_{q}$ of the *q*-th sub-tree is set as $max(abs\left(found\left(k^{\prime }\right),found\left(u^{\prime }\right)\right))$, where $found(k^{\prime })$ and $found(u^{\prime })$ represents the account balance of $V_{k^{\prime }}$ and $V_{u^{\prime }}$. *Q* sub-trees correspond to *Q* trade indexes $\left\{\delta_{1},\ldots ,\delta_{Q}\right\}$. The smaller the trade index, the more balanced the buyer and seller. Let $\delta_{q1}$ be the minimum of $\left\{\delta_{1},\ldots ,\delta_{Q}\right\}$, and $\delta_{B}$ be the trade index of $V_{k}$ and $V_{u}$. If $\delta_{q1}<\delta_{B}$, the seller retains the $V_{u,q1}$ with the least transaction index. The remaining tasks $V_{u}-V_{u,q1}$ are set as the trade tasks, i.e., $EV=V_{u}-V_{u,q1}$. Figure 7 shows an example of the tree-partition strategy. Alternatively, $\delta_{q1}>\delta_{B}$ indicates that the tasks of the seller and buyer do not become balanced after the task transaction. A new $v_{m}$ from AC is set as the candidate trade task EV, and the tree-partition strategy is applied for the new $v_{m}$. If all cells in AC can not provide more balance partitions, a new task transaction is performed since $V_{u}$ and $V_{k}$ are a pair of non-tradable partitions. With the tree-partition strategy, a set of tasks rather than a single task are reallocated, while keeping the connectivity of partitions.

In the third step, the buyer and seller trade tasks and update their account balances. The buyer $V_{k}$ purchases the task set EV, and its account balance becomes $w\left(V_{k}\right)-w\left(EV\right)+D_{s,k}$, where w(EV) and $D_{s,k}$ represent the sum of weights of EV and the shortest distance between the starting point $p_{s}$ and $V_{k}$. $D_{s,k}$ is calculated so the further-distance-travelling robot is compensated by assigning fewer tasks instead of dividing the region into *K* equal sections. Similarly, the seller $V_{u}$ sells the task set EV and adjusts its account balance to $w\left(V_{u}\right)+w\left(EV\right)+D_{s,u}$, where $D_{s,u}$ represents the shortest distance between the starting point $p_{s}$ and $V_{u}$.

With the completion of task transactions, The *K* partitions become more and more balanced. As soon as the number of task transactions reaches the preset upper limit, the partition refinement component ends and returns *K* partitions $\left\{V_{1},\ldots ,V_{K}\right\}$.

### 5.3. Path Planning

**Figure 8:**
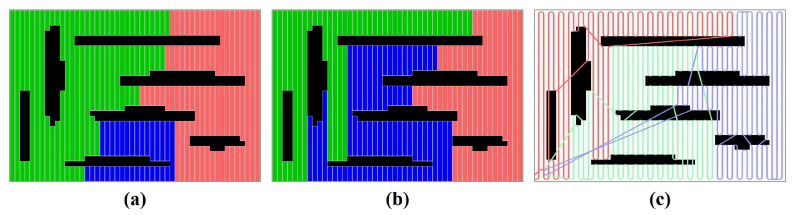
(**a**) Initial partition. (**b**) Partition refinement. (**c**) Path Planning.

After receiving *K* partitions from the partition refinement component, the path planning component applies the single-robot Dubins solver [35] to each partition. A set of *K* Dubins coverage paths is generated with each one corresponding to one robot. The complete coverage is achieved if each robot moves along the corresponding coverage path. Figure 8 shows an example of the CDM algorithm.

### 5.4. Pseudo-Code of the CDM Algorithm

Algorithm 2 shows the pseudo-code of the CDM algorithm. It decomposes the region A into a set of cells *C* and represents all cells in a graph G1 (Lines 2–3). The graph *G* is divided into *K* partitions by the initial partition (Line 4). These *K* partitions are refined by task transactions (Lines 5–23). For each task transaction, the buyer $V_{k}$ and the seller $V_{u}$ are determined, followed by the set of adjacent cells AC between $V_{k}$ and $V_{u}$ (Lines 7–8). The seller $V_{u}$ is the partition that is adjacent to and can trade with $V_{k}$. For each cell in AC, the tree-partition strategy calculates the corresponding trade tasks EV (Line 11). If EV≠∅, $V_{k}$ and $V_{u}$ trade tasks and updates their account balances (Lines 13–14). The symbol succeed, which indicates the success of the task transaction, is marked as true (Line 15). If succeed remains false, $V_{k}$ and $V_{u}$ are marked as a pair of non-tradable partitions (Lines 19–21). Upon the number of task transactions equalling MaxI, *K* partitions $\left\{P_{1},\ldots ,P_{K}\right\}$ is obtained. Next, the single-robot Dubins solver [35] is used for each partition to generate coverage paths $\left\{P_{1},\ldots ,P_{K}\right\}$ (Lines 24).
**Algorithm 2** CDM Algorithm**Input**: A,R, $p_{s}$**Parameter**: MaxI: The maximum number of task transactions
**Output**: $P_{1},\ldots ,P_{K}$
  1:  Initialize: $P_{1},\ldots ,P_{K}\leftarrow \emptyset$;

  2:  C← Area_Decomposition(A);

  3:  G1← Graph_Representation($C,p_{s}$);

  4:  $found,V_{1},\ldots ,V_{K}\leftarrow$ initial_partition(G1,s);

  5:  count←0;

  6:  **while**
count<MaxI
**do**

  7:    $V_{k},V_{u}\leftarrow$ determine the seller and the buyer;

  8:   AC← calculate the set of adjacent cells between $V_{k}$ and $V_{u}$;

  9:   succeed←false;

 10:   **for** each $v_{m}$ in AC
**do**

 11:     EV← tree_partition($V_{k},V_{u},v_{m}$);

 12:     **if** EV≠∅
**then**

 13:         Trade tasks EV;

 14:         Update found;

 15:         succeed←true;

 16:         break;

 17:     **end if**

 18:   **end for**

 19:   **if** succeed=false
**then**

 20:      Mark $V_{k}$ and $V_{u}$ as a pair of non-tradable partitions.

 21:   **end if**

 22:   count++;

 23: **end while**

 24: $\{P_{1},\ldots ,P_{K}\}\leftarrow$ Dubins_Solver($\left\{V_{1},\ldots ,V_{K}\right\}$);

 25: **return**$P_{1},\ldots ,P_{K}$;


*Computational Complexity*: Let *M* be the number of cells in *C*. The initial partition component takes O(M) times. The complexity of the partition refinement component is O(M×MaxI) in the worst case, but the worst cases are scarce. The Dubins solver takes O$\left(M^{3}\right)$ times to calculate the Dubins path [38]. Thus, the overall complexity of the CDM algorithm is O$\left(M^{3}\right)$.

## 6. Experiments

The computational experiments were carried out on a PC with Intel(R) Core(TM) CPU i5-8300H, 2.30 GHz processor, 16 G RAM, WIN 10. All experiments were performed on Dubins robots with kinematic constraints such as a forward speed of 1.0 m/s and a minimum turning radius of 1 m. A task sensor with a detection range of 1 m was incorporated into each robot. First, to demonstrate the superiority of the proposed algorithms, comparison experiments with exact and heuristic algorithms were conducted on different size maps. Second, simulation experiments based on a high-fidelity UAV model [39] were conducted to verify the feasibility of EDM and CDM.

### 6.1. Comparison Experiments in Small Scenes

**Figure 9:**
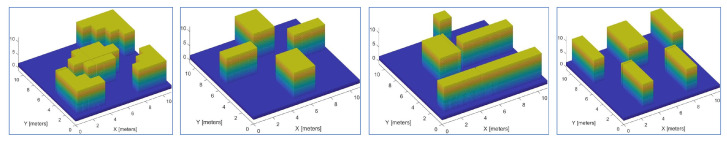
The four point-cloud maps where EDM and CDM were tested. Each environment has the size 10 m × 10 m × 10 m.

The first level of validation was performed via simulations on four small scenes with size 10 m × 10 m × 10 m. Figure 9 demonstrates the point cloud maps of four scenes, which contain several obstacles with irregular shapes and same heights. For each scene, Dubins robots start and end at the same starting point, located in the bottom left corner of the map. EDM and CDM were compared with the exact Milpflow algorithm [20] and heuristic DCRC algorithm [3]. Milpflow provides a precise area-division result instead of coverage paths. In order to achieve a fair comparison, the state-of-art Dubins solver [35] is employed to plan Dubins path for Milpflow. DCRC generates an optimal Hamiltonian path and divides the path into *K* sub-paths. Exact Mofint and EDM algorithms utilize the Gurobi optimization tool [37] to obtain the optimal solution, and their optimization time is uniformly set as 1200 s.

**Figure 10:**
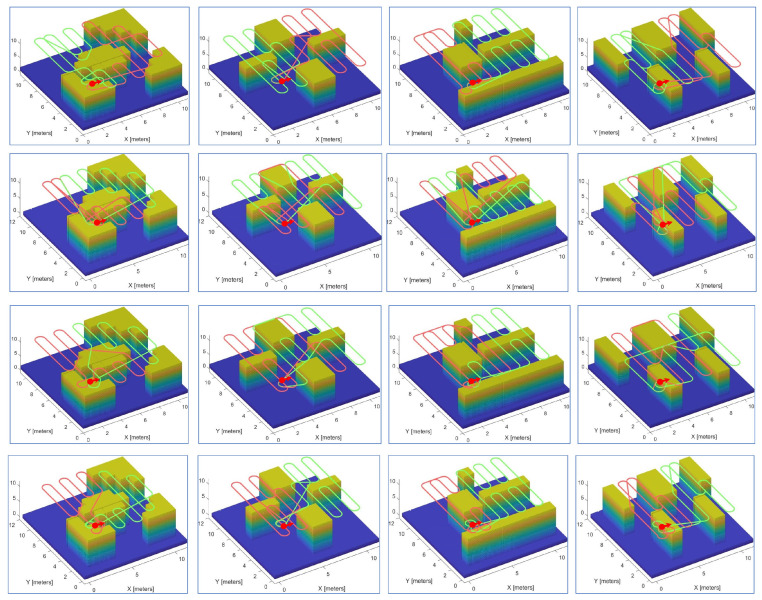
Simulation instances with two robots. The first to fourth rows represent the snapshots of the coverage paths provided by Milpflow [20], DCRC [3], EDM, and CDM, respectively.

**Figure 11:**
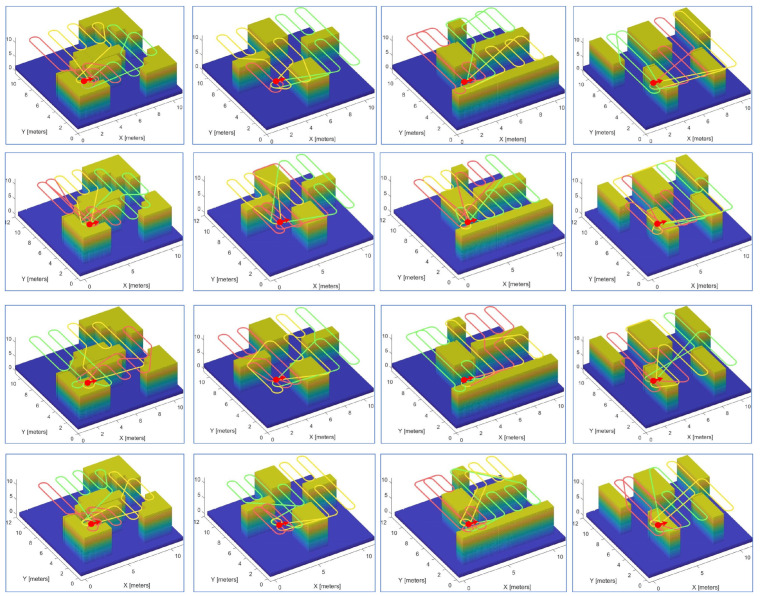
Simulation instances with three robots. The first to fourth rows represent the snapshots of the coverage paths provided by Milpflow [20], DCRC [3], EDM, and CDM, respectively.

A variety of experiments were performed using teams of two or three robots on different maps. Figure 10 and Figure 11 demonstrate snapshots of the coverage paths produced by Milpflow [20], DCRC [3], EDM, and CDM, respectively. These snapshots show that Milpflow and CDM produce relatively concentrated paths for every robot since they allocate a set of connected coverage cells to every robot. In contrast to Milpflow and CDM, EDM and DCRC generate a single-robot coverage path that is not limited in a particular area.

**Figure 12:**
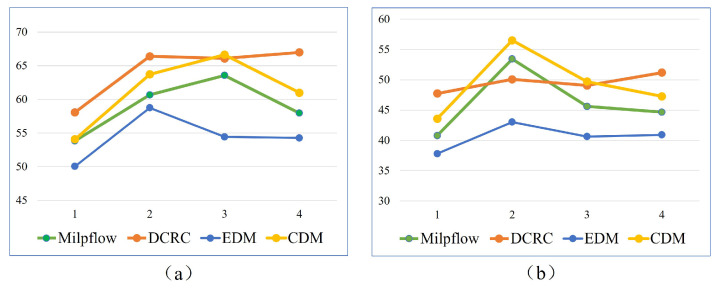
The comparison of coverage times of Milpflow [20], DCRC [3], EDM, and CDM. Fewer coverage times are better. (**a**,**b**) shows comparison results of two and three robots, respectively.

Figure 12 compares the coverage times of Milpflow [20], DCRC [3], EDM, and CDM, respectively. The comparison results show that, compared with heuristic DCRC and CDM, Milpflow and EDM provide fewer coverage times by thoroughly searching the solution space. Furthermore, EDM produces the least coverage times in all scenes because it generates the optimal Dubins coverage path rather than the area division provided by Milpflow.

### 6.2. Comparison Experiments in Large Scenes

**Figure 13:**
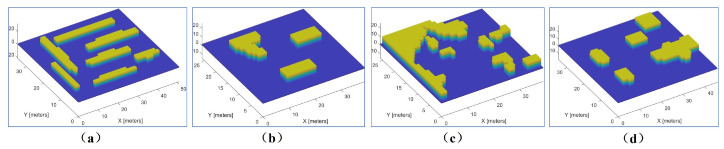
Four point-cloud maps where CDM and DCRC were tested. (**a**) Multi-cell (34 m × 50 m × 10 m); (**b**) Farm (25 × 38 m × 10 m); (**c**) Rural Quebec (25 m × 38 m × 10 m); (**d**) Cave (34 m × 45 m × 10 m).

In order to evaluate the performance of the proposed algorithm, a variant of the well-known environments from [3] was used. As shown in Figure 13, the maps differ in terms of sizes and shapes. A set of experiments were conducted with teams of {3,6,9,12} robots. Since Milpflow and EDM cannot provide efficient solutions within a limited time, this subsection only evaluates the heuristic CDM and DCRC [3] algorithms. Two metrics were used for performance evaluation as follows: (i) coverage time, and (ii) computation time.

**Figure 14:**
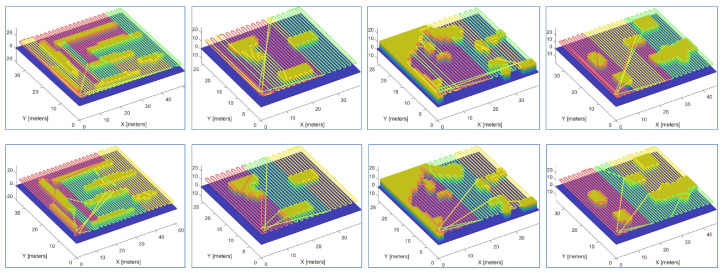
Coverage paths of DCRC (**first row**) and CDM (**second row**) with three robots.

**Figure 15:**
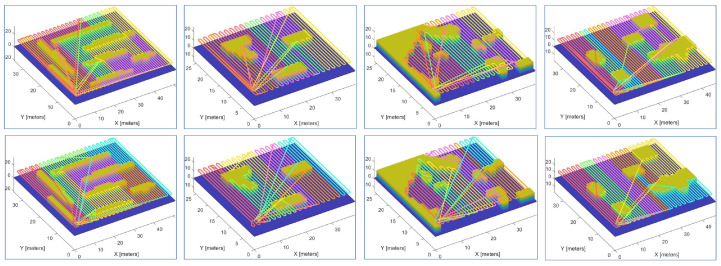
Coverage paths of DCRC (**first row**) and CDM (**second row**) with six robots.

**Figure 16:**
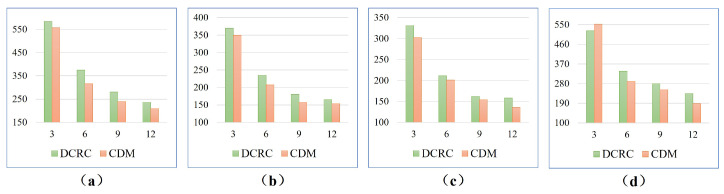
The comparison of coverage times for four different environments. Less coverage times are better. (**a**–**d**) show comparison results in multi-cell, farm, rual quebec, and cave scenes, respectively.

Figure 14 and Figure 15 demonstrate snapshots of coverage paths generated by DCRC and CDM for three and six robots, respectively. These snapshots show that the CDM algorithm provides a set of connected cells for every robot, while a single robot’s coverage cells in DCRC may be disconnected. Paths between disconnected cells probably revisit the covered area, which increases the coverage time. Indeed, as illustrated in Figure 16, the CDM algorithm provides fewer coverage times than DCRC in most experiments.

**Figure 17:**
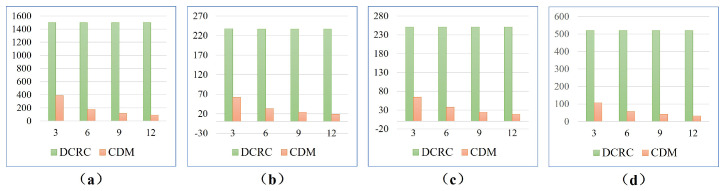
The comparison of computation times for four different environments. Less computation times are better. (**a**–**d**) show comparison results in multi-cell, farm, rual quebec, and cave scenes, respectively.

Figure 17 shows the computation times of CDM and DCRC with {3,6,9,12} robots, respectively. It is observed that DCRC provides a approximately equal computation time in each scene, while the computation time of CDM decreases with the increase in robot number. The difference in computation time between CDM and DCRC derives from the search space. The larger the search space, the longer the computation time of the algorithm. DCRC plans a single-robot coverage path in terms of the entire map, which corresponds to a large search space. In contrast, CDM divides the map into *K* sub-areas and plans the path for each sub-area. Compared with the entire map, sub-areas corresponds to a small search space. With the increase in robot number, CDM’s computation times become smaller.

### 6.3. Feasibility Experiments of EDM and CDM

**Figure 18:**
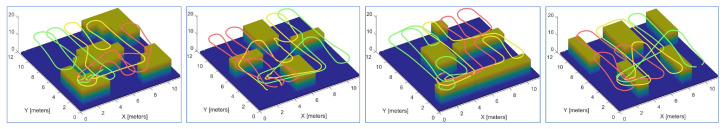
UAV simulated paths of EDM with three robots.

**Figure 19:**
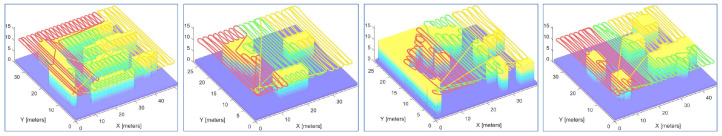
UAV simulated paths of CDM with three robots.

We validate EDM and CDM algorithms with a high-fidelity fixed-wing UAV model [39] in Simulink. A waypoint follower is integrated into the fixed-wing UAV model, which calculates the desired heading based on the current pose, look-ahead distance, and coverage paths. Experiments were conducted on UAVs with kinematic constraints, such as a 0.5 m turning radius and 1 m/s speed. Each UAV was set at a different flight height to ensure its safety. Figure 18 and Figure 19 demonstrate snapshots of the simulated UAV paths for EDM and CDM, respectively. The snapshots show that EDM and CDM are applicable to fixed-wing UAVs.

## 7. Conclusions

This paper presents an EDM algorithm and a heuristic CDM algorithm to address the Dubins MCPP problem. EDM formulates the Dubins MCPP problem into an MILP to produce the shortest Dubins coverage paths. CDM balances the coverage tasks among robots by a credit model and reduces the complexity of the Dubins MCPP problem by a tree-partition strategy providing an approximate optimal solution. It is shown that both EDM and CDM can provide smooth and continuous Dubins coverage paths. Comparison experiments with other exact or heuristic algorithms demonstrate that EDM produces the fastest Dubins coverage path in small-scale scenes, and CDM produces less coverage times and shorter computation times than other heuristic algorithms in large-scale scenes. Feasibility experiments show that the results from the simulations and the analyses performed on those results hold for high-fidelity Dubins robotic systems. Future research areas include: (i) extending online coverage to unknown environments, (ii) applying to real Dubins robots.

## Data Availability

Not applicable.
